# Effects on the Cell Barrier Function of L-Met and DL-HMTBA Is Related to Metabolic Characteristics and m^6^A Modification

**DOI:** 10.3389/fnut.2022.836069

**Published:** 2022-04-06

**Authors:** Fangrui Zuo, Hongkui Wei, Jian Peng, Shengqing Li, Yuanfei Zhou

**Affiliations:** ^1^Department of Animal Nutrition and Feed Science, College of Animal Science and Technology, Huazhong Agricultural University, Wuhan, China; ^2^Wuhan Sun HY Biology Co., Ltd, Wuhan, China; ^3^The Cooperative Innovation Center for Sustainable Pig Production, Wuhan, China; ^4^State Key Laboratory of Agricultural Microbiology, College of Science, Huazhong Agricultural University, Wuhan, China

**Keywords:** DL-HMTBA, IPEC-J2 cells, methionine metabolism, m^6^A, barrier function

## Abstract

Methionine is a substrate for protein synthesis and participates in many other biological events *via* its metabolism. We have previously demonstrated significant differences in the metabolism of L-methionine (L-Met) and its precursor DL-2-hydroxy-4-methylthiobutyric acid (DL-HMTBA) in IPEC-J2 cells. When DL–HMTBA is added to the diet, intracellular methionine (Met) sources also contain the natural form of L-Met. Then, what is the effect on Met metabolism when these two Met sources exist simultaneously? Moreover, the effects of metabolic differences on cell function remain unclear. In this study, it was found that when the proportion of L-Met to DL–HMTBA was ≤ 40%:60%, Met transmethylation was promoted and when the proportion of L-Met to DL-HMTBA was ≤ 85%:15%, Met trans-sulfuration and regeneration were improved. In addition, DL-HMTBA improved the cell barrier function when the ratio of L-Met to DL-HMTBA was ≤ 40%:60%. This finding may be due to the decrease in the proportion of *S*-adenosylmethionine to *S*-adenosylhomocysteine and mRNA *N*^6^-methyladenosine (m^6^A) levels, which increase the mRNA stability and protein expression of tight junction zona occludens-1. To sum up, the effects of L-Met and DL–HMTBA on Met metabolism, especially transmethylation, suggest that DL–HMTBA has the potential to influence the intestinal barrier function of animals through epigenetic processes.

## Introduction

The intestinal tract is the leading site for digestion and absorption of nutrients, and it is also a crucial defense barrier for the body. The intestinal epithelial layer forms a physical barrier, and its integrity and barrier properties are mainly regulated by tight junctions, which are the critical junction complex at the cell apex and primarily of zona occludens-1 (ZO-1), claudin, occludin, and other proteins (1, 2). Clinical data suggest that intestinal inflammation is often accompanied by a decline in barrier function, which is probably associated with the disruption of tight junctions (1). Methionine is an important sulfur-containing amino acid that has been shown to affect intestinal tight junction expression (3, 4). The 2-hydroxy-4-methylthiobutyric acid, another Met precursor commonly used in livestock production, has also been reported to regulate intestinal barrier function, and its effect differs from that of DL-Met (5, 6). Nevertheless, the regulatory mechanisms of these two Met sources on intestinal barrier function are not well-understood.

Methionine is a substrate for protein synthesis and participates in many other biological events *via* its metabolism. Met and its metabolites taurine (Tau) and glutathione (GSH) can exert antioxidant efficacy (7, 8). *S*-Adenosylmethionine (SAM), a metabolite of Met, is a vital methyl donor and participates in DNA, RNA, and proteins methylation (9, 10). Therefore, except for its biological function as a substrate for protein synthesis, the regulatory role of Met in metabolism is worth attention. In our prior study, we found that, unlike L-Met, DL-HMTBA significantly affected the contents of SAM and *S*-adenosylhomocysteine (SAH) and the proportion of SAM to SAH in IPEC-J2 cells (11). SAM is known as a major methyl donor in the body, and the proportion of SAM to SAH is considered a metabolic indicator or predictor of cell methylation potential (12, 13). Hence, the functional differences of Met sources may be related to their metabolic influence on methylation status.

IPEC-J2 cells, porcine intestinal cells isolated from the jejunum of newborn piglets, are helpful for characterizing the barrier function of the pig intestine *in vitro* and are often used to explore the influence of nutrients on intestinal health (14–18). The purpose of our research was to test whether DL-HMTBA can regulate Met metabolism differently from L-Met, and regulate epigenetics, especially the m^6^A modification of mRNA, which affects the expression of tight junctions, playing a different protective role in the intestinal barrier. Therefore, in IPEC-J2 cells, we studied the influence of different proportions of Met sources on barrier function, compared the metabolic differences of Met sources and the correlation between metabolites and the expression of function-related genes, and explored the effects of L-Met and DL–HMTBA on the m^6^A modification of ZO-1 after comparing the methylation status of DNA and RNA.

## Materials and Methods

### Materials

Methionine, 5-methylthioadenosine (MTA), cystathionine (Cysta), cysteine (Cys), SAM, homocysteine (Hcy), SAH, GSH, 5-methyltetrahydrofolate (5-MTHF), vitamin B6 (VB6), vitamin B9 (VB9), vitamin B6 (VB12), acetonitrile, formic acid, methanol, ascorbic acid, ammonium acetate, tris-(2-carboxyethyl) phosphine, hydrogen peroxide (H_2_O_2_), 3-(4,5-dimethyl-2-thiazolyl)-2,5-diphenyl-2H-tetrazolium bromide (MTT) were supplied by Sigma–Aldrich (St. Louis, USA). Fetal bovine serum, antibiotics (penicillin and streptomycin), and Dulbecco's modified Eagle's medium mixed with Ham's F-12 were provided by Gibco (Shanghai, China). DL–HMTBA was offered by Macklin (Shanghai, China).

### Cell Culture

IPEC-J2 cell line was a kind gift from Dr. He Qigai (Huazhong agricultural university, Wuhan, China). Cells were used between passages 70 and 90 and the cell inoculating density was 5,000/well or 3 × 10^5^/well. Each experiment was repeated three times IPEC-J2 cells were cultured in Dulbecco's modified Eagle's medium mixed with Ham's F-12, which was supplemented with 10% fetal bovine serum (v:v) and antibiotics (penicillin and streptomycin) at 37°C in a humidified incubator with 5% CO_2_. Cells were cultured without fetal bovine serum for 18 h and then without Met for 6 h, at last, different ratios of L-Met and DL–HMTBA were given for 2 h.

### MTT Assay

Cells (5,000/well) were seeded in 96-well microplates overnight, then dealt with different concentrations of H_2_O_2_ for 24 h or different ratios of L-Met and DL–HMTBA for 2 h and then 0.8 mM H_2_O_2_ for 24 h. Then, 10 μl MTT was added to each well, and the cells continued to incubate for 4 h. The supernatant was discarded, and formazan crystals were resuspended with 150 μl dimethyl sulfoxide. Finally, absorbance was read at 490 nm by a spectrophotometer. Results were presented as a percentage of surviving cells compared with control cells.

### Trans-Epithelial Electrical Resistance (TEER) Measurements

Cells (3 × 10^5^/well) were seeded in the upper room of Transwell plates (Corning, USA), then 2.6 ml medium was added into the basal chamber. Millicell-ERS instrument (Millipore, Bedford, USA) was used to measure TEER values. The measured values in the blank wells of uninoculated cells were background TEER. The final TEER values were corrected for background and displayed as Ω cm^2^.

### Real-Time PCR Analysis

RNA from IPEC-J2 cells was extracted using TRizol reagent (Life Technologies, Merelbeke, Belgium). TaqMan Reverse Transcription Kit (Thermo Fisher, USA) was used to transcribe 2 μg RNA into cDNA. Bio-Rad CFX Connect™ Real-Time PCR Detection System (Bio-Rad, USA) was used to measure the relative mRNA expression. Results were calculated by the 2^−ΔΔCT^ method. The following primers (Supplementary Table 1) were synthesized by Sangon (Shanghai, China).

### Detection the Contents of Met-Related Metabolites and Cofactors

Impacts of different ratios of L-Met and DL–HMTBA on Met metabolites and coenzyme was measured by LC–MS/MS, the specific experimental steps were shown as described previously (11). Contents of analytes were calculated per 1 × 10^6^ cells.

### Western Blot

Cells were lysed with RIPA buffer containing protease and phosphatase inhibitors (Thermo Scientific, USA) for 30 min, the products were centrifuged for 15 min at 12,000 × *g* at 4°C, and supernatants were detected for the total protein contents. A total of 30 μg proteins were treated with 10% SDS-PAGE and then transferred to nitrocellulose membranes. They were then incubated with 5% non-fat milk for 2 h and incubated with the primary antibodies: anti-ZO-1 (1:1,000, ABclonal, #A11417), anti-FTO (1:1,000, Santa Cruz, #sc-271713), anti-YTHDF2 (1:1,000, Proteintech, #24744-1-AP), anti-METTL3 (1:1,000, Abcam, #ab195352), and anti-β-actin (1:1,000, ABclonal, #AC026). After washing three times, blots were incubated with the secondary antibody (1: 15,000): anti-rabbit or anti-mouse IgG for another 2 h. Signals were detected with chemiluminescence. Band intensities were measured by Image J (NIH, USA).

### Assay of the Fluorescent Yellow Flux Rate

After the test, the culture solution was discarded, the cells in the Transwell chamber were washed with D-Hank's solution preheated at 37°Cthen 1.5 ml of fluorescent yellow solution (100 μg/ml) was added into the upper room. Next, the Transwell plates were placed in a cell culture incubator for 2 h. After treatment, the liquid in the lower chamber was collected for testing. The absorbance was measured with a fluorescence spectrophotometer, the excitation and emission wavelength of which were 427 and 536 nm, respectively. The ratio of fluorescent yellow contents in the lower and upper room was the fluorescent yellow flux rate (%).

### ELISA Assay for H_2_S

Cells were lysed with RIPA buffer containing protease and phosphatase inhibitors (Thermo Scientific, USA) for 30 min at 4°C, the products were centrifuged for 15 min at 12,000 × *g*, 4°C, and then the supernatants were collected to determine total protein content. Concentrations of H_2_S in the remaining supernatant were determined using ELISA Kit (Camilo, Nanjing, China). Briefly, samples were incubated in the microplates, which were pre-coated with monoclonal antibodies, then a specific enzyme-linked antibody for H_2_S was added. After washing for 5 times, the substrate solution was added to develop color. Results were measured using a spectrophotometer at a 450 nm wavelength.

### ELISA Assay for m^5^C

Total DNAs from IPEC-J2 cells were extracted by using a DNA Extraction kit (Genenode, Beijing, China). MethylFlash Methylated DNA Quantification Kit (Epigentek, NY) was used for the detection of DNA methylation. Briefly, genomic DNA was denatured and then incubated with capture and detection antibodies for 5-methylcytosine (m^5^C) and results were measured using a spectrophotometer at a 450 nm wavelength. Results were displayed as the percentage of m^5^C (%) in the total cell DNA.

### Assay of m^6^A Level

Effects of L-Met and DL–HMTBA on m^6^A modification in IPEC-J2 cells were measured by LC–MS/MS. The specific experimental steps were shown as described previously (19). The calculated m^6^A/A was determined for the m^6^A level.

### An m^6^A Immunoprecipitation Real-Time PCR of the Target Genes

An m^6^A immunoprecipitation (MeRIP) was applied to detect the specific m^6^A modification levels of the target genes, the specific experimental steps were shown in the previous study (19). The primers used for this detection were shown in Supplementary Table 2.

### Date Analysis

Data were shown as the mean ± SEM and analyzed using GLM one-way ANOVA in SAS 8.2 software, and Ducan multiple comparisons. GraphPad Prism version 8.0 was used for correlation analysis. *p* < 0.05 means that the dates are statistically significant and *p* < 0.01 shows extreme significance.

## Results

### Influence of Different Ratios of L-Met and DL–HMTBA on the Barrier Function

In this study, IPEC-J2 cells were dealt with different ratios of L-Met and DL–HMTBA (100 and 0%, 85 and 15%, 70 and 30%, 40 and 60%, 0%, and 100%) with normal culture or H_2_O_2_ added. When DL–HMTBA was used as the Met source, mRNA expression levels of ZO-1, claudin, and occludin were significantly upregulated, and the expression level of ZO-1 gradually increased with the increase in DL—HMTBA proportion (*p* < 0.05) (Supplementary Figure 1).

**Figure 1:**
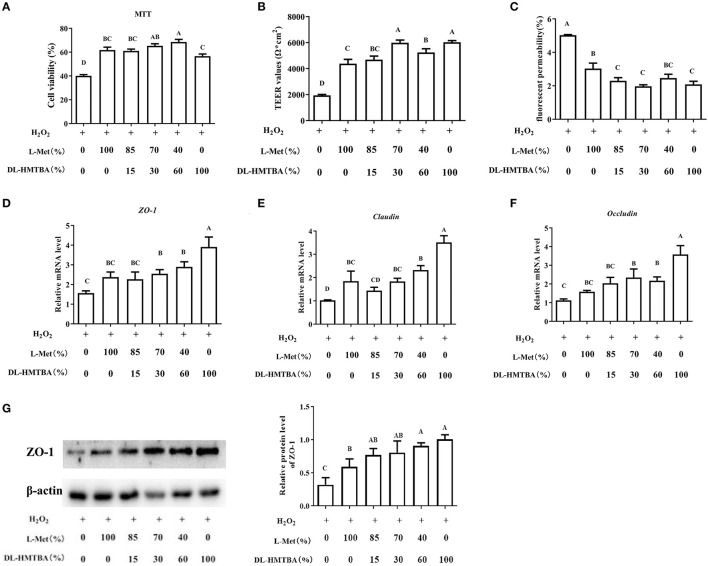
Effects of different ratios of L-Met and DL–HMTBA on the barrier function in IPEC-J2 cells. **(A)** Cell viability after Met source intervention; **(B)** TEER values; **(C)** Fluorescent yellow permeability; **(D)** mRNA levels of ZO-1; **(E)** mRNA levels of Claudin; **(F)** mRNA levels of Occludin; **(G)** Protein level of ZO-1. Cells were cultured without fetal bovine serum for 18 h and then without Met for 6 h, then, different ratios of L-Met and DL–HMTBA were given for 2 h (total methionine source concentration is 5 mM), at last cells were treated with 0.8 mM H_2_O_2_ for 24 h. Values are means ± SEM, *n* = 3; different letters (A–D) mean the difference is extremely significant, *p* < 0.01.

Then, the influence of pre-treatment of IPEC-J2 cells with different ratios of L-Met and DL–HMTBA on the permeability of monolayer cells after H_2_O_2_ injury was investigated. According to the results of the MTT assay, treatment with 0.8 mm H_2_O_2_ for 24 h was selected to build the oxidative stress model (Supplementary Figure 2). Compared with the 100% L-Met group in the presence of H_2_O_2_, when the ratio of L-Met to DL–HMTBA was ≤ 40%:60%, the cell viability was significantly higher; when the ratio was ≤ 70%:30%, the cell TEER value was significantly higher; when the ratio was ≤ 85%:15%, the fluorescent yellow permeability was significantly lower (Figures 1A–C) (*p* < 0.01). Further studies on the gene and protein expression levels of tight junctions have shown that when only DL–HMTBA is present, mRNA levels of ZO-1, claudin, and occludin were significantly higher (Figures 1D–F), and when the ratio was ≤ 40%:60%, the protein level of ZO-1 was significantly higher (Figure 1G).

### Impacts of Different Ratios of L-Met and DL–HMTBA on the Metabolism of Met

**Figure 2:**
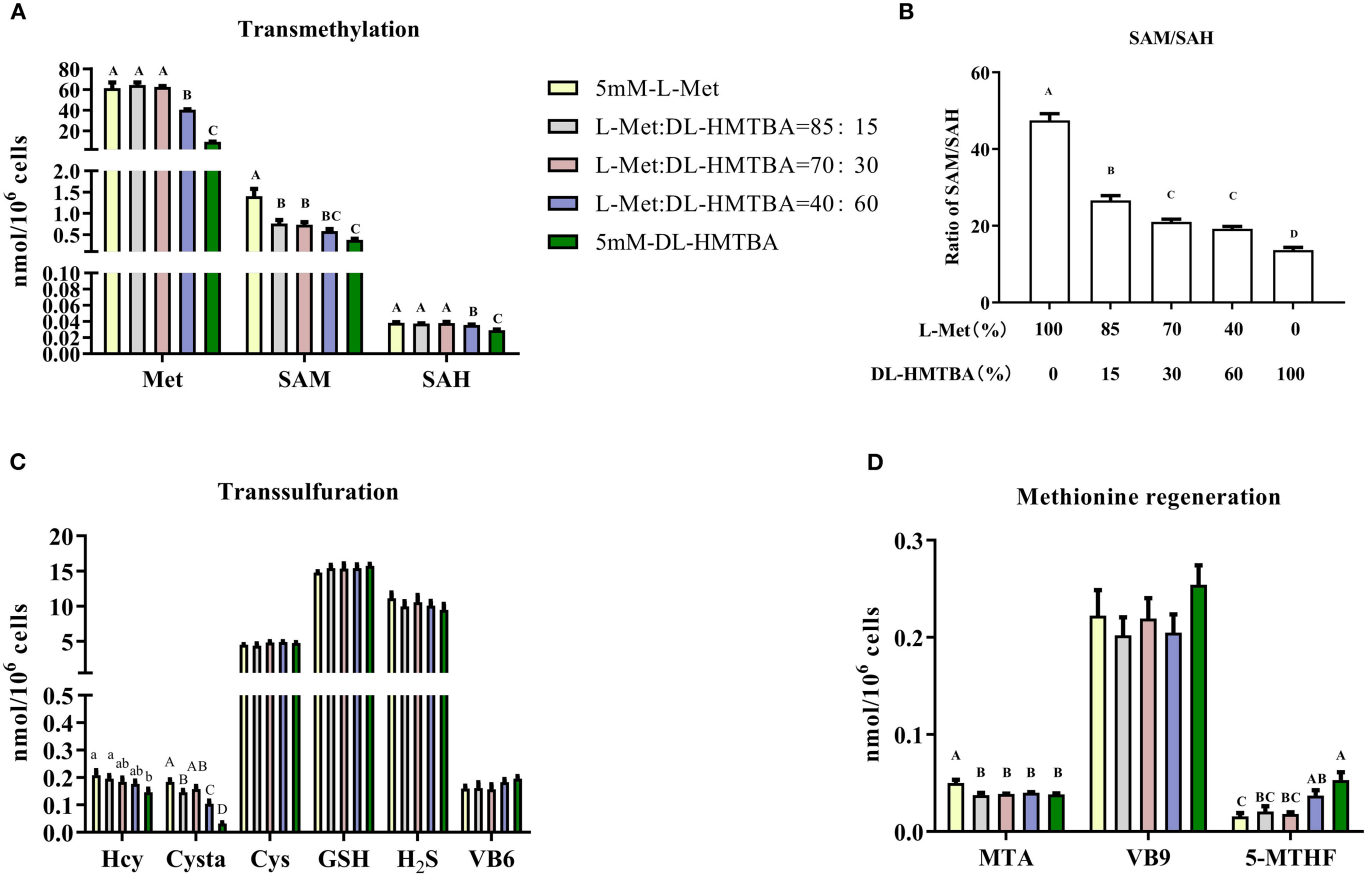
Effects of different ratios of L-Met and DL–HMTBA on the changes of methionine metabolites and coenzyme in IPEC-J2 cells. **(A)** Transmethylation-related metabolites; **(B)** Ratio of SAM to SAH; **(C)** Transsulfuration-related metabolites; **(D)** Methionine regeneration related substances Cells were cultured without fetal bovine serum for 18 h and then without Met for 6 h, then, different ratios of L-Met and DL–HMTBA were given for 2 h (total methionine source concentration is 5 mM). Metabolic pathways are shown in Supplementary Figure 4. Values are means ± SEM, *n* = 3; different letters (A–D) mean the difference is extremely significant, *p* < 0.01; different letters (a,b) mean the difference is signific, *p* < 0.05.

Next, metabolic changes of Met with different ratios of L-Met and DL–HMTBA were investigated. Compared with the 100% L-Met group, when the proportion of L-Met to DL–HMTBA was ≤ 85%:15%, the contents of SAM, Cysta, MTA, and the proportion of SAM to SAH were significantly lower; when the ratio was ≤ 40%:60%, the contents of Met and SAH were significantly lower; when only DL–HMTBA was present, the intracellular Hcy content was significantly lower (*p* < 0.05). Besides, the contents of Cys, GSH, and H_2_S and the cofactors VB6, VB9, and VB12 involved in metabolism in each treatment group were not significantly different (Figure 2).

### Influence of Different Ratios of L-Met and DL–HMTBA on the Changes of Pivotal Enzymes in the Metabolism of Met

**Figure 3:**
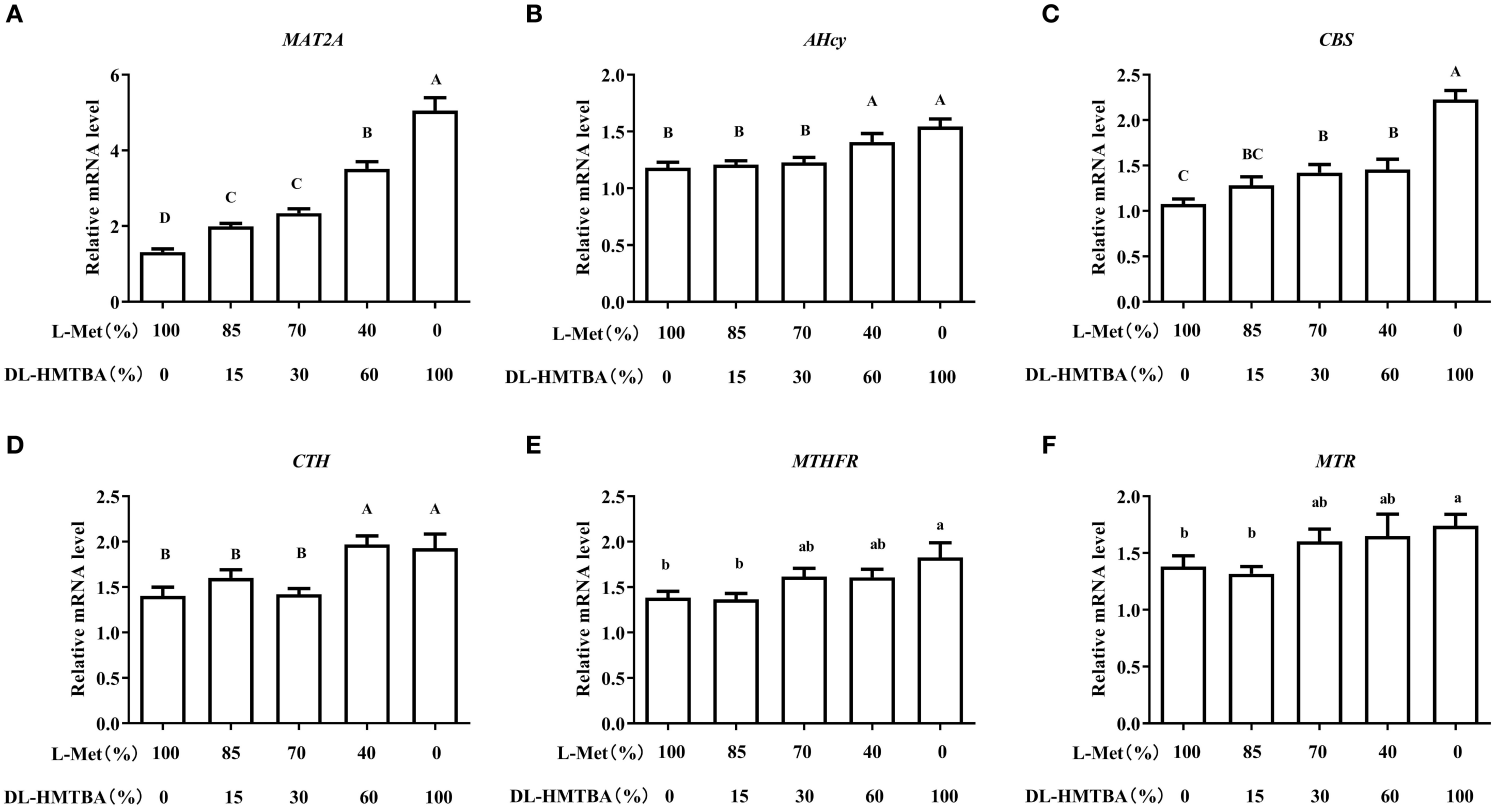
Effects of different ratios of L-Met and DL–HMTBA on mRNA levels of pivotal enzymes about Met metabolism in IPEC-J2 cells. **(A)** MAT2A: Methionine adenosyltransferase 2A; **(B)** AHcy: S-Adenosylhomocysteine hydrolase; **(C)** CBS: Cystathionine-beta-synthase; **(D)** CTH: Cystathionine gamma-lyase; **(E)** MTHFR: Methylenetetrahydrofolate reductase; **(F)** MTR: 5-methyltetrahydrofolate-homocysteine methyltransferase. Cells were cultured without fetal bovine serum for 18 h and then without Met for 6 h, then, different ratios of L-Met and DL–HMTBA were given for 2 h (total methionine source concentration is 5 mM). Values are means ± SEM, *n* = 3; different letters (A–D) mean the difference is extremely significant, *p* < 0.01; different letters (a,b) mean the difference is signific, *p* < 0.05.

We then detected changes in the mRNA levels of pivotal enzymes associated with the metabolism of Met. Compared with the 100% L-Met group, when the proportion of L-Met to DL–HMTBA was ≤ 85%:15%, the mRNA level of methionine adenosyltransferase 2A (MAT2A) was significantly higher; when the ratio was ≤ 70%:30%, the mRNA level of Cysta-beta-synthase (*CBS*) was significantly higher; when the ratio was ≤ 40%:60%, the mRNA levels of S-adenosylhomocysteine hydrolase (AHcy) and Cystagamma-lyase (CTH) were significantly higher (*p* < 0.01), in addition, only in 100% DL–HMTBA group, the mRNA levels of 5-methyltetrahydrofolate-Hcy methyltransferase (MTR) and methylenetetrahydrofolate reductase (MTHFR) were significantly higher (Figure 3) (*p* < 0.05).

### Correlation Analysis Between Met Metabolites and Gene Expression Levels

**Figure 4:**
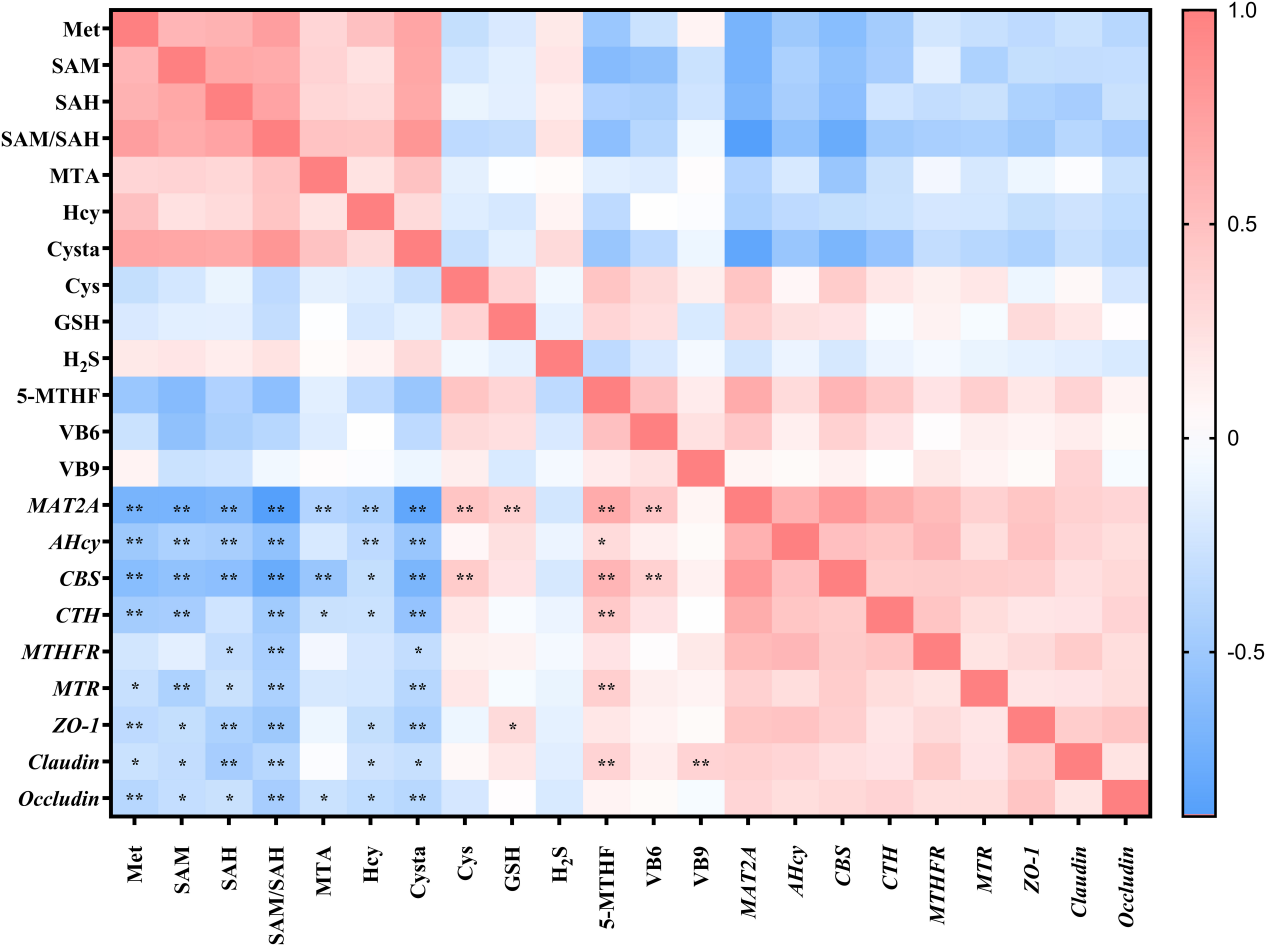
Correlation analysis between methionine metabolites and gene expression levels in IPEC-J2 cells. Cells were cultured without fetal bovine serum for 18 h and then without Met for 6 h, then, different ratios of L-Met and DL–HMTBA were given for 2 h (total methionine source concentration is 5 mM). *p < 0.05; **p < 0.01.

The aforementioned studies showed that DL–HMTBA significantly affects Met metabolism and gene expression in IPEC-J2 cells. Then, is there a specific correlation between changes in metabolites and changes in gene expression levels? Therefore, we next analyzed the correlation between metabolites of Met and the gene expression of Met metabolic enzymes and tight junction proteins. As shown in Figure 4, intracellular contents of Met, SAM, SAH, Hcy, Cysta, and the proportion of SAM to SAH, were significantly negatively correlated with the mRNA levels of most genes (*p* < 0.05). Notably, a significant negative correlation was between the proportion of SAM to SAH and each gene expression level (*p* < 0.01), and the correlation coefficient with the mRNA level of MAT2A was 0.884.

### Effects of Met Sources on DNA and RNA Methylation Modification

**Figure 5:**
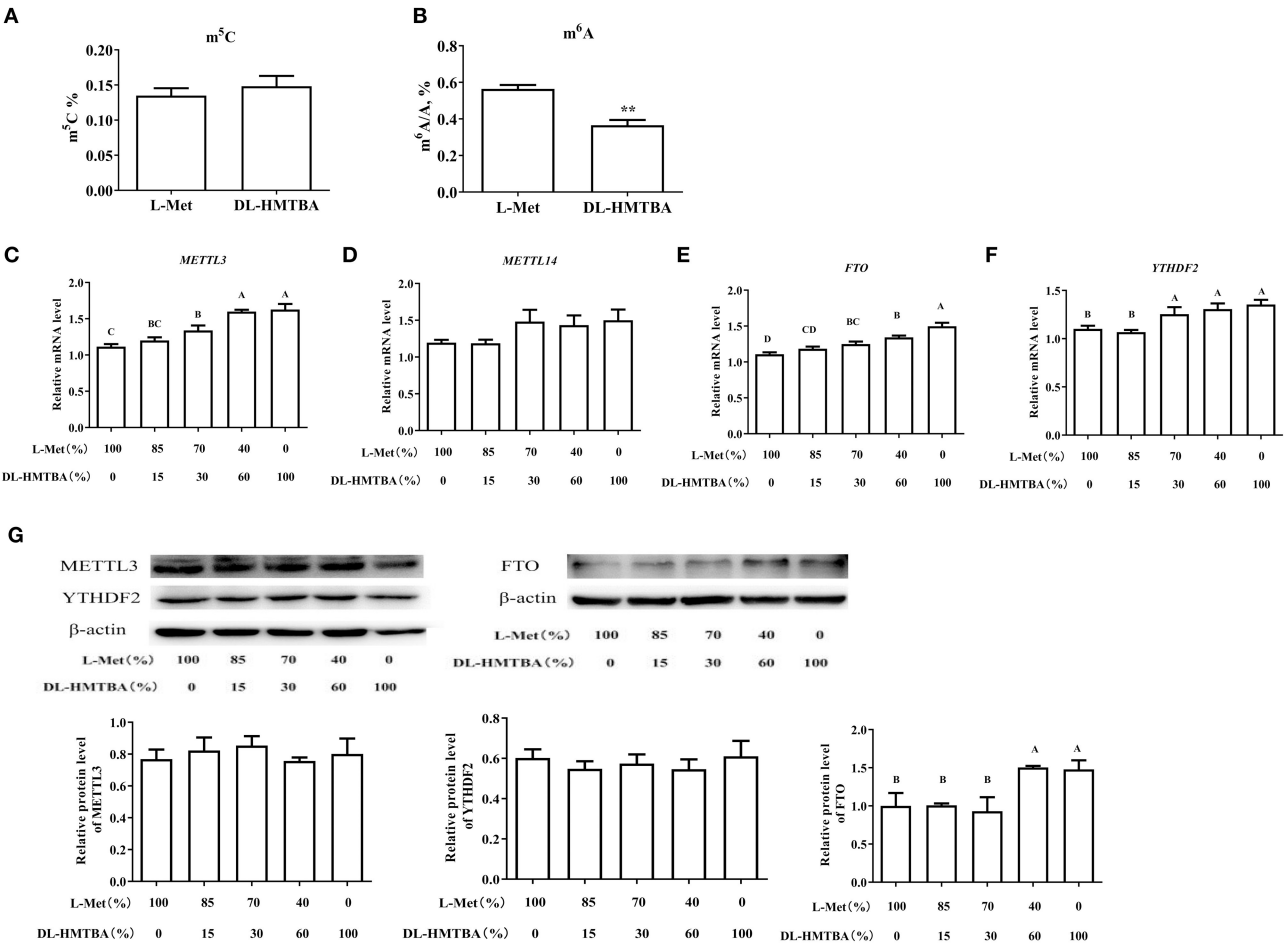
Effects of L-Met and DL–HMTBA on methylated modification in IPEC-J2 cells. **(A)** Total m^5^C level; **(B)** Total m^6^A level; **(C)** mRNA levels of METTL3 (methyltransferaselike 3); **(D)** mRNA levels of METTL14 (methyltransferaselike 14); **(E)** mRNA levels of FTO (Fat mass and obesity-associated protein); **(F)** mRNA levels of YTHDF2 (YT521-B homology domain family 2); **(G)** Protein levels of RNA methylation-related proteins. Cells were cultured without fetal bovine serum for 18 h and then without Met for 6 h, then, different ratios of L-Met and DL–HMTBA were given for 2 h (total methionine source concentration is 5 mM). Values are means ± SEM, *n* = 3; different letters (A–D) mean the difference is extremely significant, *p < 0.01; **p < 0.01.

We considered that the difference in mRNA expression levels caused by DL–HMTBA and L-Met might be due to the difference in the metabolism between them, which leads to the difference in the contents of SAM and SAH and then changes in intracellular methylation modification. Therefore, we next tested the contents of m^5^C and m^6^A, the major methylation modifications of DNA and mRNA, respectively. As shown in Figure 5A, L-Met and DL–HMTBA had no significant effect on m^5^C content in the cell DNA, whereas DL–HMTBA significantly reduced m^6^A mRNA levels (*p* < 0.01).

Therefore, we examined the mRNA levels of the major RNA MTRs, demethylases, and recognition proteins and further detected the protein levels of the genes with significant changes. As shown in Figures 5C–G, when the ratio was ≤ 70%:30%, the mRNA levels of MTR-like 3 (METTL3), fat mass and obesity-associated protein (FTO), and YT521-B homology domain family 2 (YTHDF2) were evidently higher than those in 100% L-Met group (*p* < 0.01). Among those rations, different ratios had no obvious impact on the protein level of METTL3 or YTHDF2. However, when the ratio was ≤ 40%:60%, the protein level of FTO was obviously higher than that in the 100% L-Met group (*p* < 0.01).

### Effects of L-Met and DL-HMTBA on the mRNA Stability and m^6^A Modification Level of Differentially Expressed mRNA in IPEC-J2 Cells

**Figure 6:**
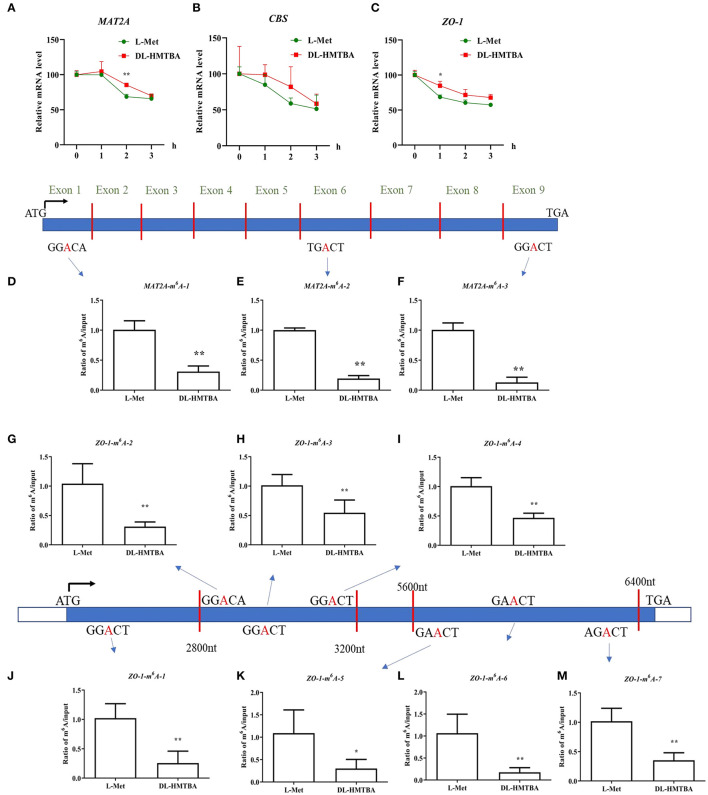
Effects of L-Met and DL–HMTBA on the mRNA stability and their m^6^A modification level of differentially expressed mRNA in IPEC-J2 cells. **(A)** mRNA stability of MAT2A; **(B)** mRNA stability of CBS; **(C)** mRNA stability of ZO-1; **(D)** The m^6^A modification site 1 of MAT2A; **(E)** The m^6^A modification site 2 of MAT2A; **(F)** The m^6^A modification site 3 of MAT2A; **(G)** The m^6^A modification site 1 of ZO-1; **(H)** The m^6^A modification site 2 of ZO-1; **(I)** The m^6^A modification site 3 of ZO-1; **(J)** The m^6^A modification site 4 of ZO-1; **(K)** The m^6^A modification site 5 of ZO-1; **(L)** The m^6^A modification site 6 of ZO-1; **(M)** The m^6^A modification site 7 of ZO-1; Cells were cultured without fetal bovine serum for 18 h and then without Met for 6 h, then, different ratios of L-Met and DL–HMTBA were given for 2 h (total methionine source concentration is 5 mM). Values are means ± SEM, *n* = 3; **p < 0.01, *p < 0.05.

L-Met and DL–HMTBA had significantly different impacts on the mRNA levels of MAT2A, CBS, and ZO-1. The aforementioned findings also showed that DL–HMTBA could reduce m^6^A modification of mRNA in IPEC-J2 cells. DL–HMTBA may affect the metabolic fate of mRNA in IPEC-J2 cells, for example, affecting the stability of mRNA. Therefore, actinomycin was used to investigate the influence of L-Met and DL–HMTBA on the mRNA stability of these genes. The results showed that the mRNA stability of MAT2A after 2 h and ZO-1 after 1 h of actinomycin treatment was significantly improved by DL–HMTBA (Figures 6A,C) (*p* < 0.05).

Next, the m^6^A modification levels of these specific differentially expressed genes, MAT2A and ZO-1, were studied. As shown in Figures 6D–M, DL-HMTBA significantly reduced the m^6^A modification levels of MAT2A and ZO-1 compared with L-Met. This finding indicated that DL-HMTBA promoted the mRNA expression of MAT2A and ZO-1, which was related to the reduction of the m^6^A modification of these two genes and the improvement in the stability of the corresponding mRNA in IPEC-J2 cells.

## Discussion

The integrity of intestinal barrier function is an important guarantee of normal physiological functions of the intestine. In this research, we found that under oxidative stress, compared with L-Met, when the ratio of L-Met to DL–HMTBA was ≤ 40%:60%, it is more conducive to the expression of intestinal tight junction proteins and the improvement of intestinal barrier function. Moreover, we found that this finding may be due to the regulation of DL–HMTBA on Met transmethylation, which may reduce the m^6^A modification level of ZO-1 mRNA and improve its mRNA stability in IPEC-J2 cells.

The influence of different ratios of L-Met and DL–HMTBA on metabolism of Met were compared, and the impact of DL–HMTBA on Met metabolite content was significantly different from that of L-Met. In our study, Met sources had a substantial influence on Met transmethylation; particularly notable is that the percentage of SAM to SAH decreased significantly with the increase in DL–HMTBA proportion. The ratio of SAM to SAH is considered a metabolic indicator or predictor of cell methylation potential (12, 13). Moreover, results displayed that the proportion of SAM to SAH was noteworthy negatively correlated with the expression of the tight junction-related genes. Results in our study indicated that the protective function of DL–HMTBA on the intestinal barrier probably be due to the regulation of methylation of genes related to barrier function. Notably, subsequent studies showed DL–HMTBA significantly reduced the m^6^A modification level of ZO-1 and improved its stability of ZO-1.

An m^6^A methylation modification is a dynamic process, and FTO is the first demethylase identified (20). In this study, it was found for the first time that with an increase in the DL–HMTBA proportion, the mRNA and protein expression levels of FTO up-regulated obviously. Therefore, the effect of DL–HMTBA and L-Met on m^6^A levels may be related to their regulation on FTO expression. However, the mechanism by which DL–HMTBA affects FTO expression requires further study.

A prior study showed that dietary Met restriction improves colon barrier function by increasing the abundance of claudin-3 (4). Epigenetic reprogramming was shown to be involved in Met restriction-related benefits (21). In our research, it was found that the barrier function and expression level of tight junction protein ZO-1 were significantly improved when the ratio of L-Met to DL–HMTBA was ≤ 40%:60%. We also found that regulation of RNA m^6^A may contribute to the DL–HMTBA-induced expression of tight junction proteins. In further research, it is interesting to explore the effect of DL-HMTBA inclusion in the diet on intestinal barrier function in an animal model.

In Caco-2 cells, it was reported that HMTBA could increase the production of Tau and reduce GSH, which is thought to be related to the more pronounced protective role of HMTBA than DL-Met on intestinal epithelial barrier function under the inflammation model (9). However, results in our study did not show any effect of Met sources on GSH content. This phenomenon may be connected with the use of different concentrations of Met sources in these experiments. In addition, there was no significant difference in the contents of Cys, GSH, and H_2_S among the treatment groups. However, Hcy and Cysta decreased with the increase of HMTBA, and the further metabolites showed no significant difference in each group. Combined with the influence of DL–HMTBA on the mRNA expression of *CBS* and *CTH*, it can be seen that DL–HMTBA can promote the transsulfuration of Met.

Except for focusing on the direct metabolites of Met, we also examined metabolites involved in the synthesis of Met. Compared with the L-Met group, despite the decrease in Met levels, the contents of 5-MTHF increased significantly after DL-HMTBA treatment, and the mRNA expression levels of *MTHFR* and *MTR*, the key enzymes involved in Met remethylation metabolism, were also significantly higher. It was found that Met deficiency increased the activity of BHMT enzyme and mRNA levels of MTR and BHMT in the liver of piglets treated with Met deficiency and supplemented with different Met remethylation donors. In addition, methyl-donor supplementation after Met deficiency makes the body more inclined to maintain the availability of Met to participate in protein synthesis (22). This indicates that DL–HMTBA can maintain the Met required for cell growth by promoting the remethylation of Met, although the content of Met produced by DL–HMTBA is low in IPEC-J2 cells.

In conclusion, we explored a new possible mechanism for the different protective effects of DL–HMTBA and L-Met on the intestinal barrier. DL-HMTBA significantly promoted Met transmethylation in IPEC-J2 cells, reducing the proportion of SAM to SAH and affecting the m^6^A modification level of cell mRNA, especially the m^6^A modification of ZO-1. Additionally, the mRNA stability and protein level of ZO-1 were upregulated, and the barrier function of intestinal cells was promoted. Upregulated FTO may contribute to m^6^A methylation modification of mRNA by DL–HMTBA. Our research indicated that the rational use of HMTBA in food might have a positive regulatory effect on intestinal barrier function and clarified possible mechanisms conducive to the reasonable selection of Met sources.

## Data Availability Statement

The original contributions presented in the study are included in the article/Supplementary Material, further inquiries can be directed to the corresponding author.

## Author Contributions

HW, JP, and FZ contributed to the conception and design of this study. FZ contributed to the experiment, analysis, and the first draft of this manuscript. HW and FZ contributed to the modification of this manuscript. SL contributed to the guidance for the detection of metabolites. All authors contributed to the article and approved the submitted version.

## Funding

This research was supported by the Hubei province technology innovation special major project (2019ABA081) and China Agriculture Research System of MOF and MARA.

## Conflict of Interest

FZ was employed by Wuhan Sun HY Biology Co., Ltd. The remaining authors declare that the research was conducted in the absence of any commercial or financial relationships that could be construed as a potential conflict of interest.

## Publisher's Note

All claims expressed in this article are solely those of the authors and do not necessarily represent those of their affiliated organizations, or those of the publisher, the editors and the reviewers. Any product that may be evaluated in this article, or claim that may be made by its manufacturer, is not guaranteed or endorsed by the publisher.
