# Supplementary material for: Effects on the Cell Barrier Function of L-Met and DL-HMTBA Is Related to Metabolic Characteristics and m6A Modification
Source: Front Nutr. 2022 Apr 6;9:836069. doi: 10.3389/fnut.2022.836069 (PMC9020446; doi:10.3389/fnut.2022.836069)
Supplement: Supplementary file 1 [file Table_1.pdf]

Table S1 Primer sequences used for the real-time PCR analysis

Gene	Primer sequences	Product size (bp)	Tm（℃）
β-actin	F: CCAGGTCATCACCATCGG	158	60
	R: CCGTGTTGGCGTAGAGGT		
MAT-2A	F：CACTTTGCCTTGGTTACGCC	85	52
	R：TCTGATGGGAAGCACAGCAC		
AHcy	F: CGGACACTTTGACGTGGAGA	93	62
	R: AACAAGTAGCGGTCCACCTG		
CBS	F: TGCTCACTACGACATCACAGC	127	60
	R: GCACTTCTCCTTCAGCTTCCT		
CTH	F: GGTTCCAACATTTCGCCACG	138	61
	R: ACTCAAAACCCGAGTGCTGT		
MTHFR	F: AGACCATACTGCACATGACCTG	155	61
	R: GTAGCTGAAGCCTCCTTCCTC		
MTR	F: TTGGAGGATGCTGTGGTA	159	55
	R: TAACGAAGTTGGTGTATGGT		
ZO-1	F: CTCTTGGCTTGCTATTCG	197	54
	R: AGTCTTCCCTGCTCTTGC		
Occludin	F: GTAGTCGGGTTCGTTTCC	167	55
	R: GACCTGATTGCCTAGAGTGT		
Claudin	F：GATTTACTCCTACGCTGGTGAC	199	58
	R: CACAAAGATGGCTATTAGTCCC		
METTL3	F: CTACTCTTGTGACCTATGCTGAT	201	55
	R: GCTCCTTGGCTACTTCTGATG		
METTL14	F: TTGACATCAGAGAACTAACACC	184	52
	R: CCAGAACCACACCAGAGAA		
FTO	F: CCGATTGGTGGTGTCAA	135	52
	R: TGGCAGTCAGGATGTCA		
YTHDF2	F: CCTCCATTGGCTTCTCCTATTC	105	55
	R: CTCTCCGTTGCTCAGTTGTC		
