# Supplementary material for: Effects on the Cell Barrier Function of L-Met and DL-HMTBA Is Related to Metabolic Characteristics and m6A Modification
Source: Front Nutr. 2022 Apr 6;9:836069. doi: 10.3389/fnut.2022.836069 (PMC9020446; doi:10.3389/fnut.2022.836069)
Supplement: Supplementary file 2 [file Table_2.pdf]

Table S2 Primer sequences used for the MeRIP-QPCR analysis

Gene	Primer sequences	Product size (bp)	Tm（℃）
MeRIP GAPDH	F: TCCTGGTACGACAATGAAT<br>R: AGATGCTCGGTGTGTTG	181	50
MeRIP MAT2A-1	F: TGAACGGACAGCTCAACGG<br>R: AGACTCGGACGTGAAGAGGA	71	58
MeRIP MAT2A-2	F: CACTTTGCCTTGGTTACGCC<br>R: TCTGATGGGAAGCACAGCAC	85	60
MeRIP MAT2A-3	F: AGAATTTTGACCTCCGCCCT<br>R: AGCTGTCCCTACCAAAGTGG	102	59
MeRIP ZO-1-1	F: TAGTGTGGGTTTGCGACTGG<br>R: CTCCTTTCGGGAGGTCAAGT	188	59
MeRIP ZO-1-2	F: TGTCAGCCCCAGGTAGTGAA<br>R: AGGACCGTGTAATGGCAGAC	165	59
MeRIP ZO-1-3	F: CACAGATACAGAAGGCGGGG<br>R: CGCTTGTGGTGAGTAGGGAG	172	60
MeRIP ZO-1-4	F: CCTCCCTACTCACCACAAGC<br>R: TTCAGGCGAAAGGTAAGGGC	120	59
MeRIP ZO-1-5	F: CGTCGGGTGATCCTAAAACCT<br>R: GAGTGGTTCCATTTCGATTTAGT	174	59
MeRIP ZO-1-6	F: AGCTGTGTCTGAACTGATGAGG<br>R: TCCAGCAACACCAGAAGCTC	105	60
MeRIP ZO-1-7	F: TAGGGAAGGACACCAAAGCA<br>R: AAGGCATTCCTGCTGGTTAGT	100	59
