# Supplementary figures and images for: Effects on the Cell Barrier Function of L-Met and DL-HMTBA Is Related to Metabolic Characteristics and m6A Modification
Source: Front Nutr. 2022 Apr 6;9:836069. doi: 10.3389/fnut.2022.836069 (PMC9020446; doi:10.3389/fnut.2022.836069)

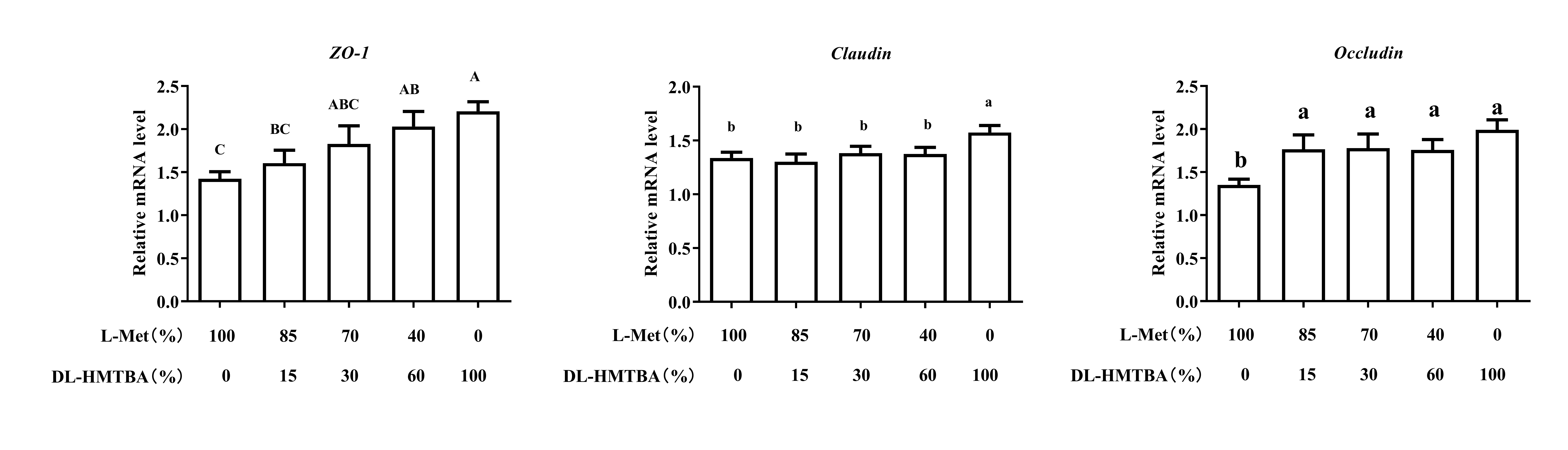

Supplement: Supplementary file 3 [file Image_1.tif]

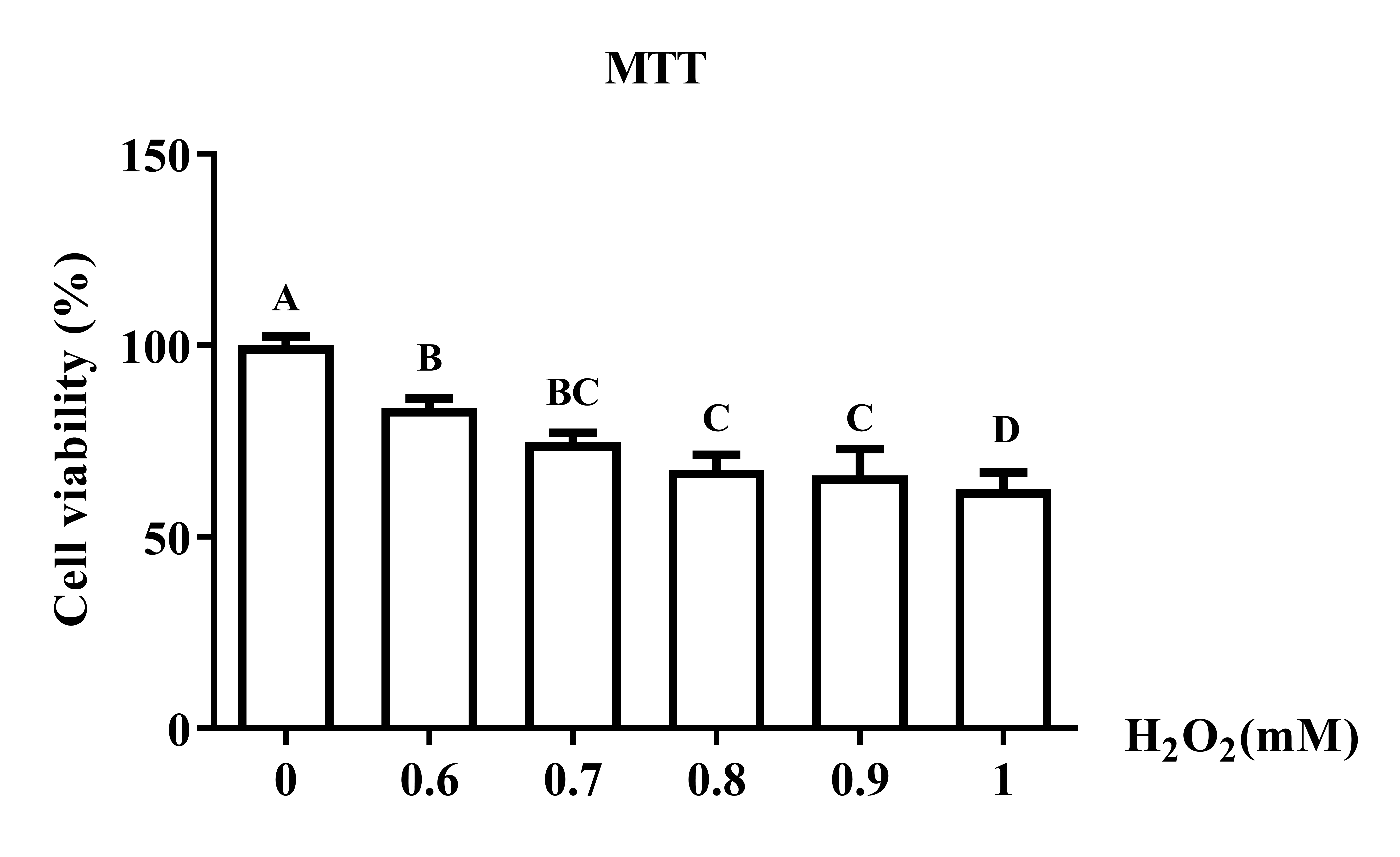

Supplement: Supplementary file 4 [file Image_2.tif]

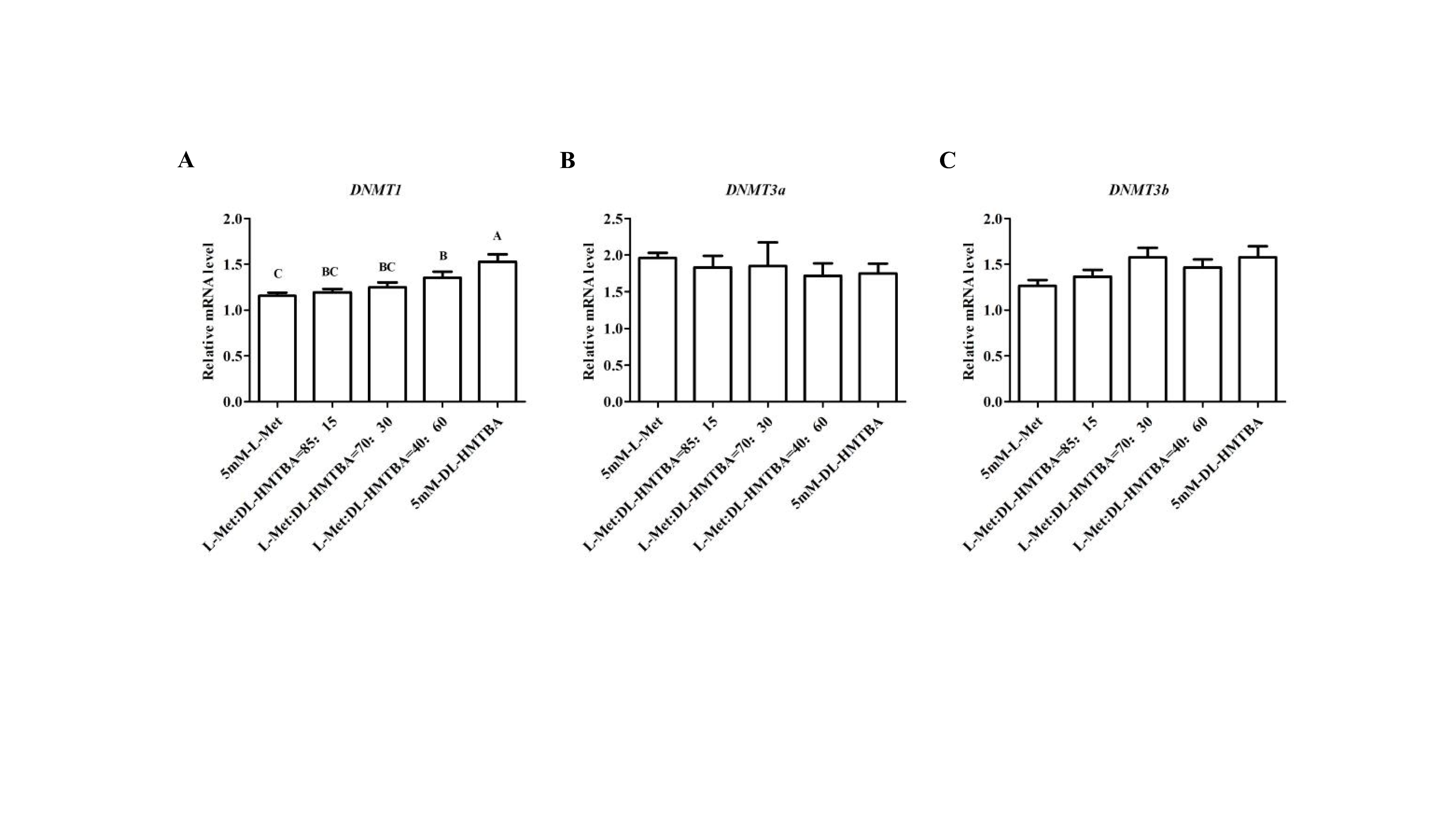

Supplement: Supplementary file 5 [file Image_3.tif]

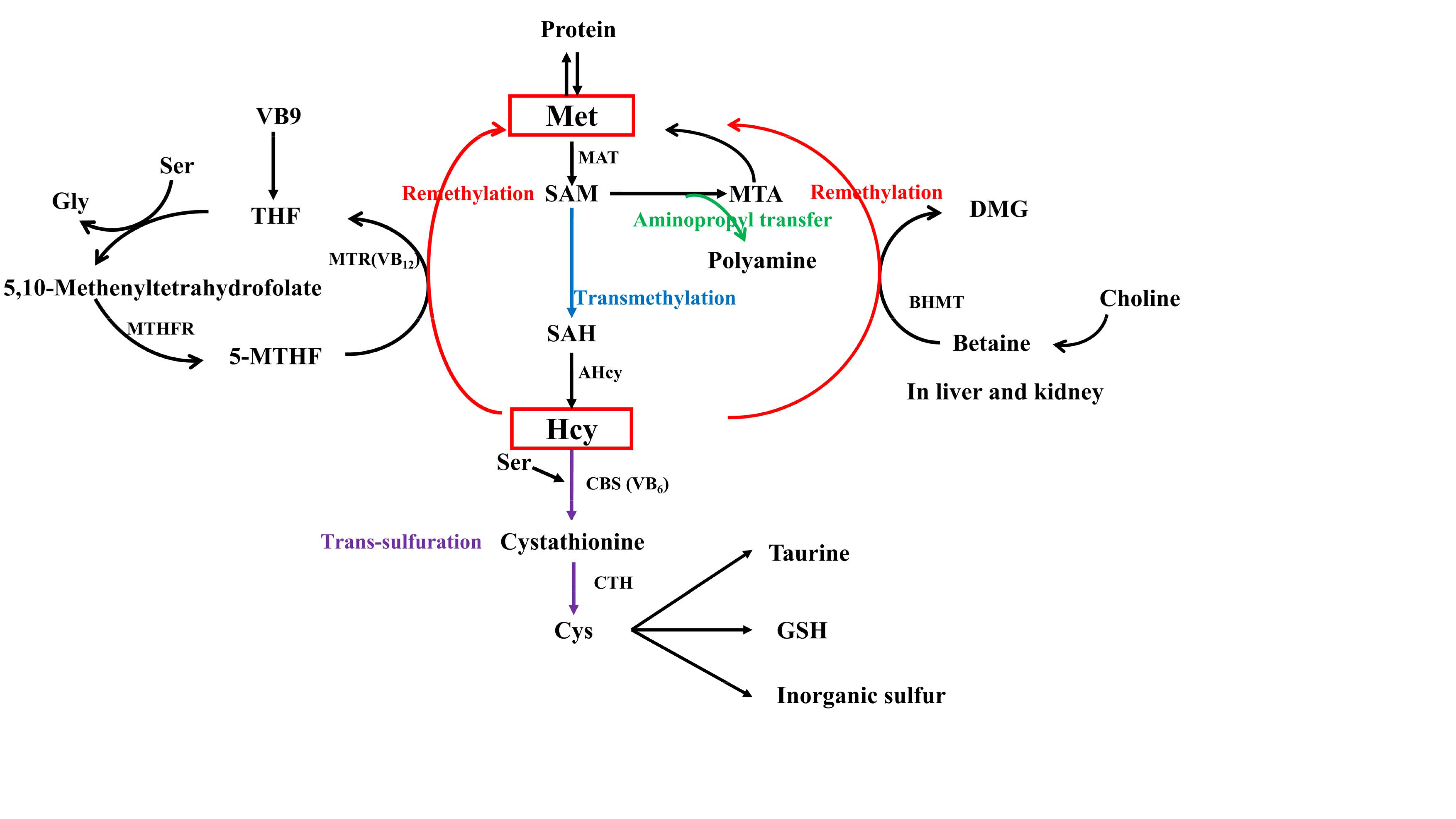

Supplement: Supplementary file 6 [file Image_4.tif]
